# Emerging predictive biomarkers for novel therapeutics in peripheral T-cell and natural killer/T-cell lymphoma

**DOI:** 10.3389/fimmu.2023.1068662

**Published:** 2023-01-26

**Authors:** Daniel Ren Yi Yap, Jing Quan Lim, Dachuan Huang, Choon Kiat Ong, Jason Yongsheng Chan

**Affiliations:** ^1^ Division of Medical Oncology, National Cancer Centre Singapore, Singapore, Singapore; ^2^ Lymphoma Genomic Translational Research Laboratory, Division of Cellular and Molecular Research, National Cancer Centre Singapore, Singapore, Singapore

**Keywords:** tumor microenvironment, genomics, checkpoint immunotherapy, precision oncology, targeted therapy

## Abstract

Peripheral T-cell lymphoma (PTCL) and natural killer/T-cell lymphoma (NKTCL) are rare subtypes of non-Hodgkin’s lymphoma that are typically associated with poor treatment outcomes. Contemporary first-line treatment strategies generally involve the use of combination chemoimmunotherapy, radiation and/or stem cell transplant. Salvage options incorporate a number of novel agents including epigenetic therapies (e.g. HDAC inhibitors, DNMT inhibitors) as well as immune checkpoint inhibitors. However, validated biomarkers to select patients for individualized precision therapy are presently lacking, resulting in high treatment failure rates, unnecessary exposure to drug toxicities, and missed treatment opportunities. Recent advances in research on the tumor and microenvironmental factors of PTCL and NKTCL, including alterations in specific molecular features and immune signatures, have improved our understanding of these diseases, though several issues continue to impede progress in clinical translation. In this Review, we summarize the progress and development of the current predictive biomarker landscape, highlight potential knowledge gaps, and discuss the implications on novel therapeutics development in PTCL and NKTCL.

## Introduction

The peripheral T cell lymphomas (PTCL) consist of several uncommon, heterogeneous subgroups of non-Hodgkin lymphomas classically associated with an aggressive clinical course and dismal survival outcomes (1). PTCL originates from post-thymic lymphocytes and span across a broad range of morphologic and immunophenotypic variability, having since been classified by the World Health Organization into 27 distinct subtypes based on clinicopathologic features and immunohistochemistry. Its most recent 2016 Fourth Edition classification of lymphoid neoplasms aids in further establishing homogeneity within these entities with the addition of recent literature (2).

Out of the PTCL subgroups, the commonest subtypes include peripheral T cell lymphoma, not otherwise specified (PTCL-NOS), anaplastic large cell lymphoma (ALCL), angioimmunoblastic T cell lymphoma (AITL), and extranodal natural killer T-cell lymphoma, nasal type (NKTCL). This classification aims to stratify disease based on the principles of its individual cell biology as well as ensuring homogeneity in the diagnostic, prognostic and therapeutic implications of disease within its overarching entity (3).

Patients with PTCL often present with advanced disease at diagnosis staged according to the Lugano classification for non-Hodgkin’s lymphoma, which is based upon the Ann Arbor system (4). Studies have shown that the majority of disease presents with stage III or IV at diagnosis, with low 5-year overall survival (OS) rates and dismal risk scores on various prognostic indices (1, 5–7). These patients also often exhibit resistance to standard chemotherapy regimens, and frequently become refractory to standard combinations indicated for the general subset of non-Hodgkin lymphomas (7, 8). When this occurs, this substantial subset of recurrent/relapsed disease subsequently fare unfavorably on second line or salvage therapy under their respective clinical trials, of which there may be no standard of care available (9, 10).

With the influx of targeted therapies in this age of molecular therapeutics, an exploration into novel treatment modalities *via* the identification of exploitable biomarkers offers a glimpse into future avenues for therapy in a landscape historically scathed by a paucity of effective therapeutic options. Despite the advent of methods such as gene expression profiling and the breakthroughs in high-throughput immunophenotyping, the identification of feasible targets remains elusive. In this Review, we thus summarize the progress and development of the current predictive biomarker landscape, highlight potential knowledge gaps, and discuss the implications on novel therapeutics development in PTCL and NKTCL.

## Search terms

In this Review, we incorporated a literature review on the MEDLINE and EMBASE databases. Search terms incorporating ‘Peripheral T Cell Lymphoma’ and its related variants, alongside ‘biomarkers’ and its related variants were used and standardized for both databases. A further in-depth search query was then applied for every identified biomarker target in conjunction with ‘Peripheral T Cell Lymphoma’. Search filters were applied to the results to include only English papers and to restrict search to articles within the last fifteen years to minimize the utilization of outdated data.

We placed an increased focus on newer papers and literature which specific respect to predictive novel biomarkers which have been identified as an exploitable immune marker for the specific treatment of peripheral T cell lymphoma. A reference of the exact search terms utilized can be found under Annex A.

## Current clinical systems for risk stratification

Risk stratification is an integral part of cancer management which guides principles of therapy and informs medical providers of prognosis for the purposes of highlighting at-risk patients for increased supervision and for end-of-life planning.

The first indicator on prognosis is conveyed right at the time of diagnosis. The exact PTCL histotype itself inherently confers prognostic information, given that certain subgroups such as ALK-positive ALCL have been shown to confer better outcomes, whilst others such as NKTCL perform considerably worse (11). With further workup, phenotypic cell markers have historically acted as surrogates for the prediction of mortality risk. One such marker used for prognostication involves Ki-67, an indicator for cell proliferation, with a higher Ki-67 staining at variable cut-offs reported by several studies to be predictive of poor outcomes (12–14). A high serum lactate dehydrogenase (LDH) level, as well as the presence of tumor Epstein-Barr Virus (EBV) infection assessed by EBV-encoded RNA (EBER) positivity, had also been identified to be predictors of worse prognosis (13, 15, 16). Interim PET/CT imaging, typically performed in the setting of Hodgkin lymphoma, has also been applied in the setting of T cell lymphomas with optimistic results on its prognostic value. Several early studies have reported that PET positivity after 2 cycles of chemotherapy indicates significantly poorer prognosis (17–19). Most recently, POD24 negativity, defined as a patient not experiencing disease progression within 24 months of front-line chemoimmunotherapy, was demonstrated to be a powerful predictor of good clinical outcome in AITL (20, 21). A common understanding, however, is that such individual factors are often too variable and lack sufficient accuracy to be utilized as sole predictors of prognosis.

Given the limitations of individual prognostic factors, clinical scoring systems have been devised to improve the accuracy and quality of analysis, composing of multiple prognostic indicators interpreted as a whole. The International Prognostic Index (IPI), initially developed for the general prognostic grading of all non-Hodgkin lymphomas, was found to be valid when applied to PTCL (11, 22). In particular, the IPI was found to be predictive of disease outcomes and each independent variable in the scoring system was shown to have significant prognostic value (23, 24). We must, however, bear in mind that this prognostic scoring system was not devised with the specifics of the PTCL cohort, and thus inevitably suffers from inaccuracy when applied to PTCL subgroups with especially optimistic or poor prognostic outcomes such as cutaneous ALCL or extranasal NKTCL, respectively (24, 25). Another subsequent limitation of the use of the IPI scoring system in PTCL is that the IPI only accurately delineated outcomes with statistical significance when stratified into two groups (simplified 2-class IPI) which simply reflected subsets of lower and higher risk (13, 24).

To better define the clinical outcomes of PTCLs as a unique entity with notable differences to other non-Hodgkin lymphomas, a separate prognostic model has been created specifically to rate outcomes of this uncommon disease. Gallamini et al. devised the Prognostic Index for PTCL (PIT) model which stratified patients into four risk categories, which held superior predictive capacity to the IPI when considering all four PIT risk classes as well as when using a simplified 2-class PIT which grouped scores of 1-2 and 3-4 into low risk and high risk groups, respectively (26). Went et al. further extrapolated on this study and proposed a modified version of PIT (m-PIT) which was the first scoring system to include the tumor-specific molecular factor Ki-67 instead of relying on the presence of bone marrow involvement, of which this modified system indeed fared better on statistical review (13). Recent additions of newer prognostic scoring systems such as the International Peripheral T-Cell Lymphoma Project Score (IPTCLP score) (14) and the T-cell score (27) which aim to identify novel prognostic markers have also contributed to the literature. These scoring systems (IPI, PIT, mPIT, IPTCLP) were each recognized to have utility in independently predicting the risk of early death within each system’s definition of intermediate- and high-risk patients derived from a cohort of predominantly Caucasian patients. Within these systems, the IPTCLP scoring system was identified to be the most valuable in predicting OS (28). We however also note that an analysis of PTCL outcomes as part of the “Intercontinental cooperative non-Hodgkin T-cell lymphoma prospective registry study in Asia” (ICT study) comprising of an Asian population yielded no significance for any of the four scoring systems in predicting survival outcomes, suggesting possible variability in the disease characteristics within PTCL of Asian origin (29).

With the influx of clinical prognostic scoring systems, further studies have also led to the creation of prognostic indices relevant to specific subtypes of PTCL itself, resulting in further specialized scoring systems. The Prognostic Index for AITL (PIAI) (30) and AITL score (31) were created to further reflect and categorize outcomes across the dismal survival outcomes of the AITL landscape; similarly, the Korean Prognostic Index (KPI) (32), Prognostic Index of Natural Killer Lymphoma (PINK/PINK-E (33), with ‘E’ representing the addition of EBV positivity data) were created for similar purposes while highlighting the varying differences in the NKTCL population. Novel prognostic models have also been suggested in recent years for NKTCL, such as the NABS score featuring the inclusion of a high peripheral blood neutrophil-to-lymphocyte ratio (NLR) of >3.5 as a scoring criterion with validated independent significance for prediction of OS and progression-free survival (PFS) (34). High initial SUVmax of the most FDG-avid lesion on primary FDG-PET/CT scanning and post-treatment Deauville scores of 4-5 which precluded a PET complete metabolic response were both additionally assessed to be associated with worse OS and PFS; a further subgroup analysis in this study further reflected that the NABS score accurately predicted survival outcomes in cohorts irrespective of the attainment of a complete metabolic response as indicated by post-treatment Deauville scores of 1-3 (35). A consolidated view of the criteria used for each prognostic index can be found below for PTCL indices and specialized indices in **Tables 1**, **2** respectively.

**Table 1:** Criteria for PTCL prognostic scores.

International Prognostic Index (IPI) (22)	Prognostic Index for PTCL (PIT) (26)	Modified PIT (m-PIT) (13)	International Peripheral T-Cell Lymphoma Project Score (IPTCLP score) (14)	T-Cell Score (27)
Age > 60 years	Age > 60 years	Age > 60 years	Age > 60 years	Albumin < 3.5 g/dL
ECOG performance status ≥ 2	ECOG Performance status ≥ 2	ECOG Performance status ≥ 2	ECOG Performance status ≥ 2	ECOG Performance status ≥ 2
LDH > ULN	LDH > ULN	LDH > ULN	Platelet count < 150,000/µL	ANC ≤ 6.5 x 10^9^/L
≥ 2 extranodal sites of disease	Bone marrow involvement	Ki-67 ≥ 80%		Stage III or IV on Ann Arbor staging
Stage III or IV on Ann Arbor staging				

LDH, lactate dehydrogenase; ULN, upper limit of normal; ANC, absolute neutrophil count.

**Table 2:** Criteria for specialized prognostic scores.

Prognostic Index for AITL (PIAI) (30)	AITL Score (31)	Korean Prognostic Index (KPI) (32) for NKTCL	Prognostic Index of Natural Killer Lymphoma (PINK) (33)	PINK-E (33) (with EBV data)	NABS Score (34)
Age > 60 years	Age ≥ 60 years	Regional lymph nodes involvement*	Age > 60 years	Age > 60 years	Neutrophil-lymphocyte ratio (NLR) > 3.5
ECOG performance status ≥ 2	ECOG performance status ≥ 2	Stage III or IV on Ann Arbor staging	Stage III or IV on Ann Arbor staging	Stage III or IV on Ann Arbor staging	Age ≥ 60 years
Extranodal sites > 1	CRP > ULN	LDH > ULN	Non-nasal primary localization	Non-nasal primary localization	Positive B symptoms
Positive B symptoms	β2-microglobulin > ULN	Positive B symptoms	Distant lymph node involvement	Distant lymph node involvement	Stage III or IV on Ann Arbor staging
Platelet count < 150,000/µL				Detectable plasma EBV DNA	

ULN, upper limit of normal; CRP, C-reactive protein; LDH, lactate dehydrogenase; EBV, Epstein-Barr virus; positive B symptoms, any one of night sweats, loss of weight, fever.

*Regional lymph nodes involvement was defined as involvement of lymph nodes corresponding to N1-N3 of the primary lesion via the TNM staging system.

The wealth of scoring systems currently available and the improvements in accuracy at predicting clinical outcomes of patients with PTCL is certainly valuable to the field. The key issue at hand, however, is that all of them are currently only validated for prognostication purposes and have yet to be fully applied or extrapolated to a predictive setting. While prognostication is indeed an important requirement in the initial workup of lymphoma, the end goal is to apply these clinical parameters towards better stratification of patients for targeted and personalized therapy, with the hope of utilizing scoring systems to guide the feasibility of treatment options such as dose intensification in the context of each individual patient to improve treatment outcomes. These scoring systems additionally do not incorporate molecular or immunopathological features, which could be instrumental in furthering the utility of such systems in the predictive context.

## The genomic and immune landscape of peripheral T cell lymphoma

PTCL is recognized as an uncommon non-Hodgkin’s lymphoma which frequently expresses pan-T cell markers such as CD2, CD3 and CD5 (6). Despite years of study, the diagnosis of specific T cell lymphoma remains challenging due to numerous overlying similarities, often requiring a consolidated review of clinical, laboratory, and immunohistological findings and expert analysis for categorization. Modern technological options such as the utility of gene expression signatures *via* transcriptomic analysis have also been studied as novel means of increasing the accuracy of diagnosing each subtype of PTCL, including further substratification of PTCL-NOS into its GATA3- or TBX21-expressing subgroups (36). This categorization was found to possibly even trump expert consensus by pathological analysis in certain cases, whereby two patients in the study were diagnosed by transcriptomic signature expression to have adult T cell lymphoma/leukemia that was previously misdiagnosed as PTCL-NOS by experts *via* pathology. Ultimately, a robust molecular classification of PTCL may be incorporated into routine clinical classification systems for improved diagnosis.

### Peripheral T cell lymphoma, not otherwise specified (PTCL-NOS)

PTCL-NOS is the most common subtype of PTCL in Western countries, constituting of a general header that categorizes T cell lymphomas that are not otherwise able to be categorized into the more distinct subtypes. As a result, PTCL-NOS as a subgroup comprises of a highly heterogeneous group of T cell lymphomas with an incompletely characterized immunophenotype and a lack of distinct clinical features. PTCL-NOS often presents as a nodal lymphoma with the potential for extranodal involvement, particularly of the cutaneous and gastrointestinal systems (37).

A gene expression model proposed by Iqbal et al. (38) has shed insight on a dichotomy in gene expression within the heterogeneity of PTCL-NOS – an expression of transcription factors T-box-21 (TBX21) and GATA 3 binding protein (GATA3) which drive differentiation of mutant T lymphocytes into Th1 and Th2 subtypes, respectively. With particular reference to the PTCL-GATA3 subgroup, the higher burden of chromosomal abnormalities as well as enrichment of *MYC* gene signatures related to proliferation (CCR4, IL18RA, CXCR7, IK), mTOR (PI3K), and marginal enrichment of β-catenin gene signature was associated with poor survival; conversely, the PTCL-TBX21 group showed significant enhancement of IFNγ-related gene signatures and NF-κB gene signatures and association with a favorable clinical outcome (38, 39). These PTCL subcategories are postulated to have evolved through distinct genetic pathways and provided biological rationale for therapeutic targets that may be exploited.

Mutations in various signaling pathways such as T cell receptors, PTEN-PI3K, NOTCH1 signaling and ITK-SYK have been postulated as progenitors of this oncogenic signaling cascade (38, 40, 41). The NF-κB pathway has also been implicated in cases of PTCL-NOS *via* identification of signals such as FYN-TRAF3IP2 (42). Mutations in *DNMT3A* within PTCL-TBX21 cases were associated with significant enrichment of activated CD8+ T-cell cytotoxic gene signatures and resulted in worse overall survival outcomes (43).

### Angioimmunoblastic T cell lymphoma (AITL)/PTCL-T follicular helper (PTCL-TFH)

A subgroup of PTCL-NOS cases was recently subclassified into a distinct subgroup as PTCL-TFH, consisting of lymphomas which were observed to manifest a T follicular helper cell phenotype including TFH-related antigens such as BCL6, CCR5, CD10. As AITL cells originate from the follicular T helper (TFH) cell, these lymphomas share considerable similarities in pathogenesis and gene expression with the AITL subgroup of PTCL (44, 45). The 2016 WHO revision acknowledged this overlap by creating ‘AITL and other nodal lymphomas of TFH origin’ as an umbrella category specifically to highlight the spectrum of lymphomas which share a TFH phenotype, given the similarities of the genetic abnormalities within these two groups, particularly that of identified pan-TFH antigens including CD279/PD1, CD10, BCL6, CXCL13, ICOS, SAP, and CCR5. The 2016 WHO classification further designates that PTCL of a TFH subtype should express at least 2 or 3 of these markers for diagnosis (2). The overarching AITL subgroup was associated with a unique spectrum of mutations consisting of *TET2*, *IDH2*, *DNMT3A*, *RHOA*, *CD28*, as well as gene fusions such as ITK-SYK or CTLA4-CD28. *TET2* mutations were present in 76% of AITL, and patients harboring *DNMT3A* mutations were found in a study to be invariably associated with the presence of a concurrent *TET2* mutation (46). *RHOA* mutations encoding a Gly17Val alteration affecting GTPase activity and inhibiting the proposed tumor suppressor function of active RHOA was identified in 53-68% of AITL cases and was similarly identified to coexist with the presence of a *TET2* mutation (47–49). In the subset of tumors harboring an *IDH2* mutation, an overwhelming majority of patients additionally presented with concurrent *RHOA* and *TET2* mutations, suggesting some interlink between the genetic pathways underlying these four mutations in AITL oncogenesis. This is further characterized by TFH lymphocyte derivation and the presence of FYN, CXCL13, PD1 and vascular endothelial growth factor (VEGF) expression in recognized AITL, along with descriptions of a proliferation of follicular dendritic cells and malignant TFH cells within close proximity high endothelial venules in pathological samples (12, 49, 50). In PTCL-TFH, *RHOA* mutations were also frequently detected, albeit at lower frequency than AITL (51).

### Anaplastic large cell lymphoma (ALCL)

ALCL is a subset of PTCL derived from Th17 cells with the characteristic expression of IL-17A and IL-17F, and is uniformly CD30-positive in nature (52, 53). The first important dichotomy in addressing systemic ALCL involves histology. ALCL is distinctly split into ALK-positive and ALK-negative subtypes with differing epidemiology, pathogenetic origin, and crucial differences in clinical disease pattern (54). ALK positivity has been recognized to be associated with significantly improved prognosis and response to first-line chemotherapy, typically anthracycline-containing regimens, additionally opening up the possibilities for novel therapeutics involving ALK-targeting agents (23). This ALK-positive subtype also expresses a unique epidemiology, characteristically presenting in pediatric patients and young adults (55). At the molecular level, ALK-positive ALCL contains a genomic rearrangement of the ALK gene on chromosome 2 with one of several partner genes – the most common being t(2;5)(p23;q35), in which *ALK* is fused with the *NPM1* gene on chromosome 5 (56).

On the other side of the equation concerning ALK-negative ALCL, a further stratification into distinct subgroups based on rearrangements of *DUSP22* and *TP63* has been associated with significant effects on prognostication. *DUSP22* rearrangement has been identified as an excellent predictor of clinical outcome similar to ALK-positive ALCL, whereas *TP63* rearrangement is instead less favorable (53). These two mutations are typically mutually exclusive and are only present in the subset of ALK-negative ALCL, whereby *DUSP22* rearrangements make up 13-30% of the cohort and *TP63* rearrangements make up 2-27% (57, 58). The remainder of ALCL that does not present with any of these alterations are subsequently dubbed as that of a ‘triple negative’ subtype with intermediate prognosis.

*DUSP22* rearrangement conferred superior five-year OS rates at 40-90% which is equivalent or even better than the previously documented ALK-positive subtype, whereas *TP63* rearrangement resulted in dismal five-year OS rates of 0-17%. The remaining ‘triple-negative’ cases retained a five-year OS of 33-42%, roughly comparable with the survival for ALK-negative ALCL before stratification into *DUSP22* and *TP63* positive subgroups (58–60). Unfortunately, these targets have not yet been exploited for targeted therapeutics, though further workup of the downstream signaling regulated by these genes could very well generate suitable targeted agents in the future.

### Extranodal NK/T cell lymphoma, nasal type (NKTCL)

Extranodal NK/T cell lymphoma, nasal type is a subset of PTCL with an aggressive clinical course and a predominance for Asian and South American populations, derived from NK or γδT cells and further characterized by frequent expansion of CD56+ and cytoCD3+ lymphocytes (2, 61). These are often generated *via* numerous pathways, of which mutations located in the *DDX3X* gene regulating RNA helicase, along with tumor suppressor genes such as *TP53* and involvement of the JAK-STAT, NF-κB, and PD-1/PD-L1 pathways stand out (62, 63). NKTCL is also always linked to positive EBV infection, expressing EBV-encoded RNA (EBER) and EBV-related proteins which could potentially be used for diagnosis and as therapeutic targets (64).

Clinically, the presence of the *DDX3X* mutation has been identified to be associated with poorer prognosis in patients treated with CHOP-based therapy (65). Given the heavy involvement of EBV in many cases of NKTCL, EBER positivity and EBV-encoded proteins such as latent membrane proteins (LMP), LMP1, and LMP2A have also been identified as characteristic of NKTCL and have potential prognostic value for NKTCL patients (66, 67). Lee et al. recently also identified EGR1 as a regulator of genes including *GAS1*, *CD59*, *CXCR7*, and *RAMP3* in NKTCL, which is often present in localized and low-risk patients and which may thus present as a tool useful for stratifying NKTCL patients who are likely to perform better (68).

### Other PTCLs

Enteropathy-associated T cell lymphoma (EATL) is classically associated with celiac disease and has been largely documented within the European continent. Its inherent association with longstanding celiac disease has caused EATL to be recognized as a complication of longstanding celiac disease, which can occur directly from a baseline of celiac disease or from a precursor, low-grade precursor intraepithelial lesion stemming from refractory celiac disease (RCD) which conveys poorer prognosis (69). RCD itself has been stratified into two distinct subtypes, with type I displaying no atypia in intraepithelial lymphocytes, T cell receptors, CD3 and CD8 expressions by intraepithelial T lymphocytes, alongside a polyclonal pattern identifiable on T cell receptor gene arrangement studies. Type II RCD (RCDII) instead displays clonal rearrangements of T cell receptors, often monoclonal, and importantly is the only subset of RCDs which present with the ability to transform into EATL (70).

Monomorphic epitheliotropic intestinal T-cell lymphoma (MEITL) was previously labelled under the header of EATL but was later revised given its non-association with celiac disease and resultant prevalence within Asia whereby celiac disease is less encountered. Mutation analyses on MEITL samples have identified involvement within *SETD2*, *TP53*, *STAT5B, GNAI2, MYC*, and *JAK3* of the JAK/STAT pathway (71, 72). CHOP-based chemoregimens were identified to be ineffective in achieving response, and thus other regimens such as ICE (ifosfamide, carboplatin, etoposide) or IVAC (ifosfamide, etoposide, high-dose cytarabine) have been pushed to the forefront of treatment for this disease. Notably, autologous stem cell transplantation (ASCT) consolidation therapy has been shown to be associated with favorable outcomes following induction chemotherapy, and has since been suggested as a current standard of care in some centers (71, 73).

Hepatosplenic T cell lymphoma (HSTL) is described as a neoplasm of mature gamma and delta T cells which infiltrate the spleen, liver, and bone marrow sinusoids of diseased individuals, often presenting with dismal survival outcomes and a predilection for a younger age of onset. Studies of HSTL specimens subjected to whole exome sequencing identified the chromatin modifying gene *SETD2* as a common mutated gene in HSTL as well, with other involved chromatin modifier genes such as *INO80, TET3, SMARCA2* and signaling pathway mutation genes such as *STAT5B, STAT3, PIK3CD*, and *TP53* being implicated. A *STAT5B* inhibitor (CAS 285986-31-4) was identified to significantly reduce cellular proliferation *in vitro* in HSTL tissue, and the addition of the *PI-3* kinase inhibitor idelalisib further reduced cellular viability in cells (74). CHOP and CHOP-like regimens have displayed lacking outcomes and for this reason non-CHOP induction followed by stem cell transplantation consolidation is the current recommendation, but the data regarding clinical trials is still presently limited in this department (75).

Daniels et al. (76) performed genome-wide sequencing on a population of primary cutaneous gamma-delta T cell lymphomas (PCGDTL), shedding insight on the nature of the origin cells of various subsets of this group of lymphomas. The PCGDTL group of cancers has been stratified into two groups originating from two distinct cancer origin cells, Vδ1, which originates from cells superficially in the epidermis or dermis and Vδ2, originating from deeper cells within the subcutaneous tissue layer. These distinct groups inform about clinical phenotype, given that Vδ1 origin T lymphomas (which includes γδ MF phenotype, γδ MF phenotype with PCGDTL-like progression, and PCGDTL phenotypes) present with much better survivals as compared to Vδ2 origin T lymphomas with a median survival of 89 months and 12.75 months respectively and present with less tumor lesions and B symptoms in patients at diagnosis; individuals with cells of origin from the epidermis (Vδ1) had significantly better survivals (179 months) as compared to those in the dermis (Vδ1, 31 months) and subcutaneous tissue layer (Vδ2, 12.75 months). The specific γδ MF phenotype only found in Vδ1 T lymphomas were also associated with better prognosis and response to therapy, though progression involving a phenotypic switch to PCGDTL resulted in a nullification of the previous survival benefits of the γδ MF phenotype subgroup.

## Present-day treatment landscape of PTCL and NKTCL

The treatment landscape of PTCL has not greatly evolved over the past decades and remains an area of unmet clinical need. In the upfront setting, the majority of patients receive CHOP-like regimens, though the exact benefit of anthracyclines remains controversial (7, 11, 77). The addition of etoposide to CHOP has been suggested to improve event-free survival in younger patients of age up to 60 years (16, 23). Outcomes of patients treated with these CHOP-based regimens are generally poor, with 5-year OS rates of less than 50% (7, 24, 77). Most trials evaluating the addition of various agents to the CHOP backbone have been disappointing, except for the use of the CD30-targeted antibody drug conjugate brentuximab vedotin in CD30+ PTCL in the pivotal ECHELON-2 trial (78). Till date, the role of consolidation with autologous stem cell transplantation remains highly debatable, though it may potentially provide benefit in patients who successfully achieve complete remission following frontline induction therapy, as well as in the subgroup of patients with AITL, advanced stage disease and intermediate to high risk IPI scores (79, 80).

In NKTCL, L-asparaginase-containing treatment regimens such as SMILE (dexamethasone, methotrexate, ifosfamide, L-asparaginase, etoposide) (81, 82), P-GEMOX (pegaspargase, gemcitabine, and oxaliplatin) (83), DDGP (dexamethasone, cisplatin, gemcitabine, and pegaspargase) (84), and AspaMetDex (pegaspargase, methotrexate, and dexamethasone) (85) have been regarded as the preferred first-line therapy for advanced disease (67). Similar to PTCL, the role of hematopoietic stem cell transplant in NKTCL is highly controversial (29, 86). Despite improvements in survival outcomes with the incorporation of these newer chemotherapeutics in the management of NKTCL, a significant proportion of patients still relapse or remain refractory to treatment. Moreover, most of these multi-agent regimens frequently risk significant adverse side effects including severe myelosuppression, infections, and hypersensitivity reactions. An optimized approach to prognostication and risk stratification, as well as the discovery of predictive biomarkers are strongly desired so as to achieve the best survival outcomes while reducing adverse effects.

In the setting of relapsed/refractory PTCL and/or NKTCL, the efficacy of contemporary salvage therapeutic options using platinum-based or gemcitabine-based regimens is typically dismal, often with response rates below 50% (29, 86). Newer chemotherapeutic agents such as bendamustine (87) and pralatrexate (8) provide only modest benefit, and strategies directed at novel targets, immune checkpoints and epigenetic pathways have been explored.

## Emerging therapeutic strategies

### Cell-surface based targeted therapeutics

#### CD52

Cell surface antigens have been long recognized as an exploitable target for various targeted therapeutics, regulating oncogenic cell signaling pathways *via* the enhancement or blockade of various cell surface molecules or utilizing them as vectors for the administration of cytotoxic material to induce tumor cell death. CD52 is a cell surface marker found on the surface of mature lymphocytes and expressed in 35-100% of PTCL-NOS, 40-100% of AITL, 25-47% of NKTCL and 0-22% of systemic ALCL patients (88–92). This receptor was first exploited by alemtuzumab, a monoclonal antibody for CD52, postulated to cause anti-tumorigenic effects through the activation of antibody-dependent cell-mediated cytotoxicity (ADCC). Whilst primarily indicated for the treatment of chronic lymphocytic leukaemia (CLL) and multiple sclerosis (MS), alemtuzumab has been explored by Gallamini et al. (93) who suggested the feasibility of first-line combination chemotherapy with a CHOP regimen combined with alemtuzumab (A-CHOP), attaining an ORR of 75%. The ACT-1 and ACT-2 trials additionally supplemented this with randomized phase III data comparing CHOP with A-CHOP in treatment naïve PTCL patients, establishing superior ORR to control although noting no significance on survival outcomes, largely attributed to treatment toxicity (94, 95). Dhanda et al. (96) also presented an observation pertaining to the change of PTCL immunophenotype from CD52+ to CD52- after alemtuzumab treatment, a phenomenon which closely alludes to the documented loss of CD20 expression after rituximab in diffuse large B-cell lymphoma (DLBCL), which is a significant cause of rituximab resistance (97). The significant toxicity and high rate of severe toxicity events preclude alemtuzumab therapy to date, and further investigations have since moved on to other therapeutic targets.

#### CCR4

CCR4 is a surface protein extensively expressed on T lymphocytes, particularly that of T-helper and T-reg cells, which as a member of the chemokine receptor family aids in cellular migration (chemotaxis) to tissue sites of inflammation (98). In diseased states, the chemokine system can be abused by tumor cells to facilitate metastasis to distant sites (99), and can directly modulate its tumor environment to enhance immune evasion (98). CCR4 was identified to be expressed in several PTCL subtypes, notably in ALCL ALK-, PTCL-NOS and AITL (100). Mogamulizumab, an anti-CCR4 antibody, targets CCR4 and induces an ADCC reaction leading to tumor cell death and lysis. A review published by Remer et al. (98) in 2014 succinctly summarized the efficacy of mogamulizumab, highlighting its feasibility in CCR4+ PTCL obtaining an ORR of up to 50%, but emphasized the high frequency of adverse events in treated populations specifically pertaining to neutropenia and lymphopenia. A multicenter phase II trial for mogamulizumab conducted in Japan further identified an ORR of 34% in their cohort of PTCL treated with the anti-CCR4 antibody, but reported no significant correlation between CCR4 expression levels and response rates to mogamulizumab. The significant frequency of severe neutropenia and lymphopenia events were further recognized by this trial, with 19% and 73% of the cohort experiencing these adverse effects respectively (101). The significant toxicity of mogamulizumab as a therapeutic option precluded its utility in achieving survival benefits and it has since fallen out of favor.

#### CD30

Brentuximab vedotin (BV) is an antibody-drug conjugate targeting CD30 as a means of internalizing the antimitotic agent monomethyl auristatin E (MMAE) for oncolysis (102). CD30 is expressed in approximately 32-64% of PTCL-NOS, 43-63% of AITL, 100% of systemic ALCL and 46-80% of NKTCL, reflecting a biomarker with significant prevalence and thus utility in the population of PTCL (14, 103–105). In part, trials evaluating the efficacy of BV together with a combination chemotherapy protocol incorporating cyclophosphamide, doxorubicin and prednisolone (BV-CHP) in CD30+ PTCL (defined as PTCL with >10% CD30 expression) has shown impressive clinical outcomes, particularly championed by the ECHELON-2 landmark trial which boasted a hazard ratio of 0.66 and 0.71 against standard CHOP therapy in terms of OS and PFS respectively, together with a reported 70.1% and 51.4% 5-year OS and PFS (78, 106). The notable outcomes and responses to this regimen has resulted in the establishment of a new standard of care for CD30+ PTCL, and has reinvigorated the field towards a cautious but optimistic search for future novel predictive biomarkers and therapeutic targets. Ongoing trials are evaluating frontline treatment using BV-CHP in PTCL with less than 10% CD30 expression (NCT04569032), as well as a similar BV-CHEP regimen which includes the addition of etoposide (NCT05006664).

#### CD25

CD25, also known as the interleukin-2 receptor α-chain or IL-2R, is positively expressed in 40-50% of PTCL (107, 108). Denileukin diftitox, a fusion protein linking diphtheria toxin to IL-2, elicited an ORR of 65% when used in combination with CHOP chemotherapy in a cohort of newly diagnosed PTCL alongside well-tolerated side effect profiles (109); applied to a cohort of R/R PTCL, it was able to elicit an ORR of 61.5% in CD25+ patients and 45.5% in CD25- patients (110). This was furthered by a phase II trial of E7777 in Japan, which features a similar mechanism of action and drug profile to denileukin difitox albeit with claims of improved purity – this new drug featured an ORR of 41.2% with one patient in the study obtaining a complete response (111). In particular, patients with less than 20% observed CD25+ cells were still able to attain an ORR of 25.0% against a ORR of 41.7% in the subgroup of patients with >20% CD25+ cells, implying that while CD25 positivity might factor in as a predictive biomarker for therapeutic response to anti-CD25 pharmacological agents, the lack of CD25 expression does not necessarily preclude E7777 as a therapeutic option in these patients. A further phase I trial of camidanlumab tesirine, an antibody-drug conjugate exploiting CD25 to deliver cytotoxic crosslinking of cellular DNA, whereby preliminary results similarly suggest correlation of CD25 expression to the drug’s therapeutic efficacy (112).

### Immune checkpoint inhibitors

Another initial area of exploration in the field of immunotherapy involves the usage of immune checkpoint inhibitors which block pathways that downregulate the immunological surveillance and induce a state of anergy in T cells. The blockade of these inhibitory signals re-activates T cells, thus mounting an immunologic response against cancer cells and inducing a phenomenon of adaptive oncologic clearance (113). Two pathways have been studied extensively pertaining to this underlying principle.

#### PD-1/PD-L1

One of the major immune checkpoint pathways involves PD-1, expressed on T regulatory (T-reg) and activated effector (CD4 and CD8) T cells and its ligand PD-L1, often expressed on evading cancer cells. When activated, PD-1/PD-L1 binding suppresses native T cell activation and by extension, adaptive T cell mediated cancer cell clearance (113). The activation of the PD-1/PD-L1 pathway has also been proposed to be mediated by the JAK-STAT oncogenic pathway activation in a subset of patients (114). High levels of PD-1 expression measured on T-reg cells in PTCL were thus identified to be associated with poorer prognosis in a group of patients (115) and recently, PD-L1 expression alongside V-domain immunoglobulin suppressor of T cell activation (VISTA) in NKTCL has been found to act as synergistic negative predictors of prognosis, further establishing the role of T cell suppression in oncologic immune evasion (116, 117).

Various immune checkpoint inhibitors have since been commissioned targeting this immune pathway. Pembrolizumab, an anti-PD-1 antibody, has been increasingly used in the literature surrounding malignancies such as melanoma and other non-Hodgkin lymphomas and has been explored in recurrent/relapsed (R/R) mature PTCL with an ORR of 33% (118). Geptanolimab and nivolumab, which are also anti-PD-1 antibodies, have been studied in R/R PTCL with an ORR of 40.4% and 33%, respectively (119, 120). In NKTCL, blockade of the PD-1/PD-L1 axis has emerged as a promising treatment strategy as well (121–124). Recent studies also noted that chidamide, a histone deacetylase (HDAC) inhibitor, seemed to leverage upon anti-tumor effects provided within PD-1+ cells to enhance the expression of genes associated with chemokine and chemotaxis activity, exposing the possibility of synergistic effects when combined with standard PD-1 blockade therapy (125, 126).

#### CTLA-4

Another major immune checkpoint pathway related to the principle of T cell anergy concerns the CTLA-4 ligand which acts as a direct and complete blockade of co-stimulation in T cells *via* its stronger affinity for B7 (CD80, CD86) as compared to the normal activator CD28 (127). CTLA-4 inhibitors thus function by inhibiting CTLA-4, allowing CD28-B7 crosslinking and activation of T cells for its activity against tumor cells. A study of PTCL *via* Sanger sequencing identified that CTLA-4/CD28 fusion genes occurred in approximately 30% of PTCL samples, and up to 58% in AITL (128). Despite the similarities that it shares with the PD-1/PD-L1 pathways, CTLA-4 inhibitors have not yet been adequately studied in the population of PTCL within clinical trials despite there being pharmacological agents such as ipilimumab available in the market for the treatment of other malignancies (128). However, a case series performed on PTCL tumors had found substantial CTLA-4 mutations in various subtypes of PTCL, notably in that of AITL and PTCL-NOS, suggesting possible room for therapeutics against the CTLA-4 surface marker in future studies (129).

### Epigenetic targeted agents

Epigenetic therapeutics involve the targeting of changes which occur in the cancer epigenome, whereby oncogenesis often is derived from changes in expression of proteins through complex factors involving the transcription and translation of genetic material *in vivo*. Two key mechanisms are understood to play key roles in the genesis of cancer through this route, histone modification and DNA methylation, which have been targeted through HDAC inhibitors and DNMT inhibitors as respective pharmacological agents.

Histone deacetylases (HDAC) are a family of enzymes which are involved in the modification of histones, predominantly deacetylating them, resulting in increased binding of DNA to histones which results in silencing and suppression of DNA transcription. HDAC inhibitors therefore aid to induce cell cycle arrest, differentiation, or apoptosis as valuable effectors of oncolysis (130). Within this class of drugs, romidepsin, belinostat and chidamide have since been trialed as monotherapy agents in the setting of R/R PTCL. They have been demonstrated in various phase II trials to induce modest responses in some patients with generally tolerable side effect profiles, with romidepsin, belinostat and chidamide exhibiting an ORR of 25%, 26%, 28% and inducing CR in 15%, 11%, and 14% of patients respectively (131–133). Interestingly, chidamide was identified to exhibit more durable responses in the population of AITL, achieving an ORR of 50% and a CR of 40% in this particular subgroup. Most recently, romidepsin was trialed together with oral 5-azacytidine in a population which included both treatment-naïve and R/R PTCL with an ORR of 61% and CR of 43%; in the subgroup analyses of treatment-naïve patients and PTCL-TFH an even higher response rate was observed, with ORRs of 70% and 80% respectively (134). However, a recent phase III study by Bachy et al. failed to find any significant improvement in PFS, OS, nor overall response rates in a comparison between romidepsin and CHOP versus CHOP-only therapy, whilst the romidepsin and CHOP group exhibited an increased incidence in severe treatment-related adverse events (135). This perhaps highlight the heterogeneity of PTCL and suggests the need for clinical trials specifically evaluating epigenetic-targeted agents in AITL/PTCL-TFH.

DNA methyltransferases (DNMTs) are a family of enzymes involved in epigenetic methylation of the human genome, which is known to result in the silencing of tumor suppressor genes; DNMT inhibitors have thus been utilized to induce hypomethylation of the genome for therapeutic benefit in cancer treatment (136). Due to the frequent involvement of the *TET2*, *IDH2* and *DNMT3A* genes in AITL subtypes which are associated with cytosine methylation and hydroxymethylation, DNMT inhibitors such as 5-azacytidine have been evaluated in AITL with reported significant and durable responses (137–139). Despite this, the recently published final results of the ORACLE phase III study comparing oral 5-azacytidine to investigator’s choice in R/R AITL failed to achieve significance in the primary endpoint of PFS with a median PFS in the 5-azacytidine group of 5.6 months versus 2.8 months in the standard arm (p = 0.0421) (140). Overall survival, however, was associated with significantly improved OS in the 5-azacytidine arm, with a median OS of 18.4 months in the 5-azacytidine group versus 10.3 months in the standard arm (HR 0.557, 95CI 0.323-0.961). Another important takeaway was that the study found no significance between the presence of *TET2*, *RHOA*, *DNMT3A*, and *IDH2* mutations and PFS or OS during subgroup analysis. The authors concluded that errors made in view of their overly optimistic hypothesis of PFS improvement might have resulted in an underpowering of the study; despite this, the favorable safety profile of oral 5-azacytidine and attainment of OS endpoints suggest utility in the treatment of AITL.

### Other immunoregulatory agents

Lenalidomide is a thalidomide analogue which has immunomodulatory activity *via* the induction of apoptosis of tumor cells and anti-angiogenic effects and is a therapeutic option often used in multiple myeloma and non-Hodgkin lymphoma. Lenalidomide has been evaluated as a possible therapeutic option in PTCL with highly variable reports of its efficacy. Multiple phase II trials of lenalidomide monotherapy in populations of PTCL involving PTCL-NOS, ALCL, and AITL yielded ORR rates of 22-26%, though there was significant heterogeneity of the treatment population consisting of both R/R PTCL and untreated PTCL patients who were not candidates for combination chemotherapy (141, 142). In AITL, the study by Morschhauser et al. reported an ORR of 31%. In recent years, combination therapy involving lenalidomide has been further explored with a combination of lenalidomide with vorinostat and dexamethasone yielding an ORR of 25% in R/R PTCL (143) and a combination of lenalidomide and CHOEP (cyclophosphamide, doxorubicin, vincristine, etoposide, and prednisone) (len-CHOEP) yielding an ORR of 68% with a CR rate of 48%, although the len-CHOEP regimen had a 38% prevalence of associated serious adverse events (144). Lenalidomide combination therapy with CHOP in AITL was also explored in one study to be associated with an ORR of 47.4% and a CR rate of 43.6% (145).

Bortezomib is a proteasome inhibitor exhibiting anti-tumoral activity through inhibition of intracellular degradation of pro-apoptotic factors such as the P53 protein and hence activation of programmed cell death, which has also been used mainly in the setting of multiple myeloma. Overexpression of microRNA-187 in PTCL-NOS has been proposed to be a potential mediator in chemoresistance whilst conversely informing of susceptibility to proteasome inhibitor therapy (146). Bortezomib monotherapy has been trialed in the setting of cutaneous T cell lymphoma with an ORR of 67% (147), as well as in numerous combination therapy regimens. Bortezomib in combination with pralatrexate had an ORR of 20% in a small series of five PTCL cases (148) and bortezomib in combination with panobinostat yielded an ORR of 43% in a study of 23 PTCL cases (149). In this latter study, the AITL population yielded the best response rates as well with an ORR of 50%. Bortezomib in combination with CHOP chemotherapy was also investigated in several trials, with an ORR of 87% in the subgroup of PTCL-NOS, AITL, and ALCL in the study led by Kim et al., which was observed to be higher than that of responses to CHOP-only therapy within the literature (150). The study yielded low ORR of 40% in the subgroup of extranodal NKTCL, which was attributed by the authors to potentially be due to the frequent expression of a multidrug-resistant p-glycoprotein in extranodal NKTCL. Of note, a newer generation of orally administrated proteasome inhibitor, ixazomib, was also investigated as a monotherapy regimen in a single phase II trial which only yielded one case of response within a population of 13 patients (151). The authors concluded that further detailed studies focusing on the mechanisms conferring susceptibility or resistance to novel therapeutic strategies, including proteasome inhibition, should be considered for future personalized treatment in this domain.

Though controversial, stem cell transplantation (SCT) is often used as consolidation therapy and autologous SCT has since been regarded by some as the mainstay of therapy for optimal outcomes in PTCL following high-dose inductive chemotherapy (152–154). The principle of treatment is for the usage of high dose chemotherapy regimens on patients with subsequent rescue *via* autologous SCT, and such a consolidation therapy utilized on first complete remission (known as ‘upfront autologous SCT’) has been validated to be associated with increased OS in all stages of disease and in patients with intermediate-to-high IPI scores (80, 155). However, in the setting of R/R PTCL, the choice of autologous versus allogenic SCT is less well understood; allogenic SCT is associated with increased side effects such as transplant-related mortality (TRM) and non-relapse mortality (NRM) in transplant patients. On this topic, a systematic review and meta-analysis by Du et al. (156) comparing outcomes between autologous and allogenic SCT in R/R PTCL has since revealed no significant difference in 5-year OS and 3-year PFS between autologous SCT and allogenic SCT group used in R/R PTCL, though the 3-year OS was significantly higher in the allogenic SCT group and especially so in the subpopulation of patients who did not attain CR before transplantation. The allogenic SCT group, as expected, also attained significantly higher rates of TRM at both the 3-year and 5-year marks. The current consensus is thus that allogenic SCT yields outcomes comparable to autologous SCT in the setting of R/R PTCL with the downside of increased transfusion-related effects, although allogenic SCT could potentially remain an appropriate option in fitter or younger patients with better baselines and less comorbidities. Autologous SCT, however, should not be routinely recommended as salvage therapy later than as second-line therapy if patients have already undergone prior multiline treatment as the associated prognosis is likely to be worse (157).

## Emerging biomarker landscape in PTCL and NKTCL

The interaction between PD-1 and PD-L1 remains a complex entity as of yet not fully understood but holds considerable importance and potential in paving the way for immunotherapeutics in PTCL (158). Studies have been conducted in datasets of solid tumors, particularly those of melanoma and NSCLC types, which identified a reasonable correlation between PD-L1 expression on immunohistochemistry and response to PD-1/PD-L1 antagonistic agents (159). In these solid tumors, the expression of markers such as HLA-DR, CTLA-4, CD56 and CD45RO, with lower amounts of CD3, CD27 and CD28 were identified in responders to PD-1/PD-L1 inhibitors (160). However, no such study has been conducted in a population of lymphomas, nor has any analysis been conducted to suggest that the similar pattern is upheld in lymphomas.

We also identify *JAK* mutations as common shared mediators of tumorigenesis across the spectrum of PTCL which remain important exploitable targets for targeted therapeutics, such as the like of JAK inhibitors which generate anti-tumoral effects *via* inhibition of the JAK-STAT signaling pathway for tumor suppression. A phase II biomarker-driven study by Moskowitz et al. (161) recently demonstrated the efficacy of the JAK inhibitor ruxolitinib across various patients with R/R PTCL in attaining a primary endpoint of clinical benefit rate (CBR), defined as the percentage of the cohort which demonstrated a complete response, partial response, or stable disease lasting at least 6 months. They crucially elucidated a meaningful association between CBR and the presence of JAK/STAT mutations and *STAT3* expression, with the subgroup with identified JAK/STAT mutations achieving a CBR of 53% and the subgroup with *pSTAT3* expression ≥30% assessed *via* immunohistochemistry achieving a CBR of 45%, significantly elevated over the subgroup which did not fulfil the criteria for either of the two categories with a CBR of 13% (p = 0.02). JAK/STAT mutations and expression thus appear to be a prospective biomarker which could readily inform clinicians on the choice of therapeutic options in R/R patients, especially as we gravitate towards the setting of personalized medicine.

### PTCL-NOS

*DNMT3A* is a gene that has been documented as a progenitor of PTCL oncogenesis, whereby its involvement in the PTCL-NOS subtype is characterized by mutations within its functional domains, featuring mutations skewed towards its MTase domain and a R882H/C hotspot mutation identified within roughly 30% of the PTCL-NOS cohort (43). In extension of the previously recognized PTCL-NOS subgroups of PTCL-TBX21 and PTCL-GATA3, *DNMT3A*-mutant patients with a concurrent PTCL-TBX21 subtype were identified to have significantly inferior OS as compared to DNMT3A-wildtype PTCL-TBX21 patients; this finding was not reproduced in the cohort of PTCL-GATA3 patients. This poor prognostic significance of *DNMT3A*-mutant PTCL-TBX21 patients was further observed to negate the survival benefits of the PTCL-TBX21 subtype over that of the PTCL-GATA3 subgroup, resulting in similar survival curves observed between these two subgroups.

### AITL

AITL is characterized by specific epigenetic mutations such as *TET2*, *DNMT3A*, *IDH2*, and *RHOA*, which have been identified as genes which regulate functionality in the sequence of events required for DNA demethylation (162, 163). As a result, hypomethylating agents have been hypothesized to be able to play a significant role in the management of AITL by directly opposing the background DNA hypermethylation that occurs as a result of these characteristic mutations, thus controlling oncogenic drive and disease. It is further theorized that hypomethylating agents such as 5-azacytidine would be effective in *TET2*-mutated cancers given the significantly increased efficacy of 5-azacytidine in *TET2* mutant subgroups in the treatment of myelodysplastic syndrome (164). Treatment with the hypomethylating agent 5-azacytidine was found to induce a sustained response, achieving 75% ORR in a cohort of 12 patients with 6 patients achieving CR, although no relationship was found between the number of TET2/DNM3A/IDH2/RHOA mutations and response to treatment in this cohort (137). A more recent retrospective study on the efficacy of 5-azacytidine as salvage therapy was recently published as well and reported 60.0% ORR in patients who were able to tolerate the optimal dose of therapy (139). The identified RHOA-VAV1 signaling in AITL may also be amenable to dasatinib, a multikinase inhibitor, with a promising outcomes observed from a preliminary phase I trial in AITL (165, 166).

AITL tumors are also identified to overexpress CXCL12 in over 50% of cases. Preliminary results of a phase two study showed that tipifarnib, a selective inhibitor of the farnesyltransferase enzyme which aids CXCL12 secretion which directs T cell homing and chemotaxis, was found to have a 43% ORR and 73% clinical benefit rate (CBR) in AITL (167). In particular, the presence of the *KIR3DL2* gene variants C336R/Q386E predicted complete response to tipifarnib therapy and improved outcomes, postulated to be due to the variants’ association with low levels of CXCL5, reducing possible resistance to tipifarnib. Finally, we appraise new data identifying AITL to express a higher quantity of PD-1 and PD-L1 in both tumor cells and the surrounding immune microenvironment, along with an association of increased PD-1 and PD-L1 expression to poorer prognosis (168). Given the current enthusiasm for PD-1 inhibitors such as pembrolizumab and nivolumab, we anticipate further data on the utility of PD-1/PD-L1 expression in predicting response to these immune checkpoint inhibitors and their novel combination regimens in AITL, although recent data suggests modest activity and risk of hyperprogression in patients treated with nivolumab alone (120).

### ALCL

ALCLs, independent of their ALK status, express near homogeneous CD30 positivity which renders this subtype of PTCL amenable to therapy with the CD30-targeting antibody drug conjugate brentuximab vedotin (169).

The presence of ALK rearrangement in ALCL predicts sensitivity to conventional anthracycline-based chemotherapeutic regimens, and thus often present with superior survival outcomes amongst PTCLs (23). The unique subset of ALK-positive ALCL patients additionally presents with the potential for targeted therapeutics involving small molecule inhibitors against ALK, such as crizotinib, ceritinib and alectinib. Crizotinib has been explored in the treatment of pediatric ALK+ ALCL patients with positive efficacy and safety profiles (170), with results demonstrating its utility in inducing complete remissions in the setting of advanced, relapsed ALK-positive ALCL cases (171, 172). In a phase two study involving 12 patients with R/R ALK+ ALCL, crizotinib monotherapy resulted in a remarkable ORR of 83.3%, with 7 patients (58.3%) achieving complete response (173). Likewise, ceritinib was demonstrated to induce durable complete remission on a singular case report (174). Fukano et al. further showed a similarly high response rate of 80% in a phase two study evaluating alectinib on 10 patients (175).

In addition, the subgroup of ALK+ ALCL boasts a unique phenotype in which PD-L1 expression was detected at high rates in the tumor and microenvironment of these lymphomas, although this has not yet been correlated to response rates to PD-1/PD-L1 targeting agents (176). The PD-L1 expression on tumor cells and tumor-infiltrating immune cells were further found to have no significance on prognosis when independently evaluated as factors, although the specific subset of ALK+ ALCL with both high tumor cell PD-L1 and high tumor environment immune cell PD-L1 yielded significantly lower 5-year PFS (177). Further studies in this regard should help evaluate the role of the extended tumor microenvironment as a biomarker in PTCL, which might present a differing point of view for further analysis apart from simply targeting tumoral cells for analysis.

### NKTCL

PD-1/PD-L1 has been explored in the setting of NKTCL, with reports of PD-L1 expression in various cohorts to range between 38 to 93% (68, 178), and the usage of PD-1 inhibitors such as pembrolizumab has shown promising results in the setting of R/R NKTCL (121, 122). Unfortunately, the expression of PD-L1 has not yet shown to be correlated to treatment response to PD-L1/PD-1 inhibitors in NKTCL; subsequent papers have also suggested that EBV latency factors such as LMP1 could induce the expression of PD-L1 in NKTCL (179), and that induced PD-L1 could be a factor of resistance to immune checkpoint blockade instead, thus explaining an apparent lack of correlation between PD-L1 expression and response to PD-1/PD-L1 inhibitors (180).

In a recent study by Cho et al. (181), immunohistochemistry was performed to stratify NKTCL cases into four subgroups, separated according to tumor immune microenvironments, through quantifying the expression of CD68, CD163, CD8 and FoxP3. These four subgroups, separated into immune-tolerant, immune evasion (A and B), and immune-silenced groups, was suggested to predict response to pembrolizumab, although the dataset was too small for definitive conclusions. Sensitivity to PD-1/PD-L1 checkpoint inhibitors has also been found to be adequately predicted by exploring a 3’-UTR mutation of PD-L1 (PD-L1^MUT^) identified *via* whole-genome sequencing (WGS) in R/R NKTCL (182). All patients whose tumor harbored PD-L1^MUT^ (5/20 cases) achieved complete response to PD-1 checkpoint inhibitor pembrolizumab, demonstrating sensitivity of 100% as a biomarker of response. Interestingly, all these patients have survived for more than 3 years which is uncommon for this group of patients. The small cohorts in these studies however, present room for further analysis of these predictive biomarkers in future, larger populations.

A study analyzing a combination of anti-PD-1 antibody therapy with pegaspargase, gemcitabine, and oxaliplatin (P-GEMOX) by Cai et al. also hypothesized that the P-GEMOX regimen might aid in sensitizing tumors to anti-PD-1 immunotherapy, alongside the *DDX3X* mutation leading to anti-PD-1 drug resistance as a negative predictive biomarker (183). V-domain immunoglobulin suppressor of T cell activation (VISTA) has also been explored as a factor which predicted negative response to the usage of PD-1 inhibitors in NKTCL, and presents as a target for future therapeutics whereby VISTA blockade could be used as a tool for increasing sensitivity to anti-PD-1 options (117).

CD38 expression status was also identified to be an independent adverse prognostic factor in patients with NKTCL, with a cohort studied by Wang et al. predicting significantly reduced PFS intervals (184). The antibody to CD38, daratumumab, has also been trialed in this group of patients in a phase two study, achieving a modest ORR of 25% (185, 186). Interestingly, the ratio of CD38 to complement inhibitory proteins (CIP) was subsequently demonstrated to better correlate with antitumor efficacy of daratumumab than using either CD38 or CIP expression alone (187). The inherent positivity of CD56 in NKTCL has also been observed; although no therapeutic target to CD56 currently exists, a study in solid tumors identified that higher expression of CD56 were observed in responders to anti-PD-1/PD-L1 therapy (160). Given the high expression rates of CD56 in NKTCL, further investigation might be warranted into the clinical significance of CD56 in therapeutics.

In terms of prognostic indices, a new-generation genomic-augmented multivariate prognostic model was recently developed *via* the identification of 13 mutated genes which offered significant prognostic significance in terms of reduced OS and PFS in a cohort of 210 NKTCL tumors, of which including BCOR, KRAS, JAK3, DCC, FAS, NOTCH1, BIRC3, etc. and implicating 39% of the total NKTCL samples used in generation of the model (178). These prognostic genes had mild correlation with high-risk features as denoted *via* previous models such as the IPI, PINK, and PINK-E, and an augmentation of these prognostic indices *via* the addition of a point in the presence of one of the 13 recognized mutations resulted in improvements in the prognostic significance of the three indices for both PFS and OS. These models were rescored and renamed as IPI-G, PINK-G, and PINK-E-G respectively. Another multivariate analysis identified a 7-SNP-based classifier which was also able to predict PFS and OS between high-risk and low-risk cohorts, with the additional benefit of having clinical predictive significance in Ann Arbor stage I patients, whereby patients classified as of high-risk were documented to receive increased OS and PFS when treated with chemotherapy in addition to baseline radiotherapy (188).

The final point is a discussion on studies pertaining to biomarkers involving the JAK-STAT signaling pathways. In particular, the expression of JAK3/STAT3 signatures and the downregulation of tumor suppressor positive regulatory domain containing I (PRDM1) was identified as a prognostic biomarker, with PRDM1-/STAT3+ phenotypes expressing poor survival outcomes (189). Most recently, Dong et al. showed that *PRDM1* loss and *STAT3* mutation cooperates to promote NK cell growth (190). JAK inhibitors such as ruxolitinib, tofacitinib, or novel small-molecule STAT3 inhibitors such as Stattic might thus pose to be a potential agent for this emerging biomarker.

### EBV-associated T cell lymphomas

Epstein-Barr virus (EBV) infection has since been recognized as a major player in the pathology of numerous B lymphomas and our understanding of its role in PTCL is steadily emerging. NKTCLs are inherently characterized by EBV involvement, whereby EBV-infected cells proliferate, undergo clonal expansion and achieve immortalization of the clonal species through an of yet unknown mechanism (191). The virally encoded latent membrane protein 1 (LMP1) has been postulated to interact with the tumor necrosis factor (TNF) family of receptors, as well as inducing gene expressions which are relevant to cell growth and the cell cycle (192). In the particular setting of NKTCL, EBV is believed to present with a type II latency pattern, which encompasses EBNA1+ and LMP1+ and other EBV-related genes (193). The role of EBV in AITL however is more controversial. EBV positive B-cells have been observed in 85-95% of AITL, although its specific role in AITL pathology and oncogenesis is still unclear (194). The presence of EBV in AITL cases were largely of EBV-1 origin, expressed genes from all stages of the EBV lytic cycle, but predominantly exhibited a type II latency pattern similarly to that observed in NKTCL (194, 195). EBV infection may therefore be exploited in the treatment of PTCL and NKTCL.

In the wake of type II latency EBV positivity in infected cells, we understand that EBNA, LMP1 and LMP2 proteins are often present (196). Kalra et al. goes further to characterize the presence of BARF1, an EBV type II and III latency-associated antigen with MHC class I and II epitopes, which might present as a useful future target for immunotherapy involving CD4 and CD8 T effector cells (197). With the underlying principle of targeting EBV as a means to eradicate the source of cancer, recent studies regarding tenofovir disoproxil fumarate (TDF) and tenofovir alafenamide (TAF) show significant antiviral activity against EBV (198). HDAC inhibitors have also been identified to induce EBV reactivation, pushing EBV cells out of their latency phase into a lytic phase, which might be postulated to aid in the prevention of immune evasion and to induce the clearance of EBV from the body (199). Certainly, whilst these present attractive possible avenues for therapeutics, many more studies will have to be performed to confirm the validity of these mechanisms.

Treatment with autologous EBV-specific cytotoxic T lymphocytes (CTLs) has been explored with a subset of patients with EBV positivity. A case series involving 10 NKTCL patients successfully utilized *in vitro* expanded antigen-specific CTLs against the EBV proteins LMP-1 and LMP-2a in a cohort of patients with lymphomas expressing EBV latent proteins, including patients with NKTCL (200). This concept was extended to a cohort which had achieved complete response post-first-line chemotherapy in an effort to prolong remission post-response (201). It also remains to be seen if the basis of targeting EBV-infected tumor cells *via* expanded autologous EBV-specific CTLs would continue to hold true in a subset such as AITL whereby the role of EBV in oncogenesis is less understood.

Finally, Myint Wai et al. (202) recently described a unique group of PTCLs which resembled extranodal NKTCL but held sufficient differences in clinical outcomes and immune signaling pathways to warrant further consideration as a subgroup distinct from that of currently defined NKTCL. This unique group of patients are presently labelled as ‘primary Epstein-Barr virus (EBV)-positive nodal T/NK-cell lymphoma’ (PTCL-EBV) and were identified *via* clinicopathological features such as the primary presentation of nodal disease where the bulk of tumor is localized, lack of nasal involvement, and/or expression of a CD8+/CD56– phenotype. Notably, these lymphomas had poorer clinical outcomes, low genomic instability, an upregulation of immune pathways involving NF-κB, IFNγ, and IL6-JAK-STAT3, and downregulation of EBV miRNA when compared to classical NKTCL. Tumors classified under PTCL-EBV exhibited significantly shorter median OS (4.6 months versus 14.7 months in NKTCL, p = 0.001), and PD-L1 was also identified to be significantly upregulated in PTCL-EBV likely from upstream pathways such as NK-κB and IFNγ which induce PD-L1 expression, presenting a potential biomarker for targeted therapy. The glaring discrepancy between the aggressiveness of PTCL-EBV tumors and its low genomic instability was proposed to be due to suppression of instability by underlying NF-κB activation amidst a paucity of *TP53* mutations, which might contribute towards the low genomic instability but high aggressiveness of this tumor.

### Other PTCLs

A study by Cording et al. (203) identified mutations in the JAK1-STAT3 pathway in 100% of RCDII samples subjected to whole exome sequencing and comparative genomic hybridization. Mutations in the NF-κB pathway were subsequently identified in 90% of the samples, implicating the JAK1-STAT3 pathway and NF-κB activating mutations in RCDII lesions; these findings were further correlated with frozen biopsy analysis *via* targeted next-generation sequencing and targeted amplicon sequencing as well as the identification of increased cytokine responsiveness through these pathways. With a comparison of EATL and RCDII-transformed-EATL displaying similarities in the involvement of the JAK1-STAT3 pathway in particular, JAK inhibitors such as ruxolitinib and abrocitinib were investigated in RCDII cell lines with an inhibition of proliferation and induction of apoptosis, presenting with the possibility of some utility of JAK inhibitors in inhibiting progression of RCDII to full-fledged EATL. The proteasome inhibitor bortezomib additionally displayed STAT3 phosphorylation and growth inhibition in RCDII cell lines, opening up possibilities regarding the therapeutic value of prevention of RCDII progression in the pathogenesis of RCDII-induced-EATL.

Huang et al. (72) created a MEITL patient-derived xenograft (PDX) model which was subsequently utilized in the performance of an AI-driven digital medicine tool, the Quadratic Phenotypic Optimization Platform (QPOP), in generating predictions of response to combinations of therapeutic regimens. The combination of pimozide and romidepsin yielded high predictive efficacy against the MEITL model and further utility of this combination could be evaluated in human trials, with reductions in dose proposed to mediate the side effect profile associated with romidepsin toxicity.

### Tumor microenvironment and immune cell function

In addition to the exploration of key cellular, immunological, and genetic features as covered above, we hope to round up our holistic discussion by considering a further look at the wider tumoral microenvironment (TME) and the complex role of different groups of microenvironmental immune cells in the interplay between new generation pharmacological agents and observed clinical responses. T_reg_ cells have been identified to loosely conform to four main functional groups in the setting of lymphomas, predominantly that of suppressor T_reg_, malignant T_reg_, direct tumor-killing T_reg_, and incompetent T_reg_, of which this classification matters as the first two groups of T_reg_ correlate higher circulating numbers of T_reg_ cells with a poorer prognosis whereas the last two groups correlate higher numbers of T_reg_ cells with an improved prognosis (204). In PTCL-NOS specifically, T_reg_ cells were more frequently associated with suppressor cell function, directly contributing to the aggressiveness of the disease.

Tumor-associated macrophages (TAMs) are also key players in the lymphoma tumor microenvironment and have been postulated to create an immunosuppressive TME through the production of cytokines, growth factors, and inhibitory immune checkpoints, leading to cancer initiation and promotion, immune regulation and distant metastasis (205, 206). Whilst not yet extrapolated to the specific subset of PTCLs, paclitaxel was identified as an agent in non-Hodgkin lymphoma which was able to potentiate phagocytic capabilities of macrophages such as TAMs in CD47-targeting macrophage therapy (207). This potentially presents us with a peek into the possibility of utilizing TAMs to remodel the TME in lymphomas to induce tumoricidal effects and oncological clearance.

We finally turn to the activity of myeloid-derived suppressor cells (MDSCs) in the TME and its role in potentiating an immunosuppressive TME. MDSCs are pathologically activated neutrophils and monocytes with inherent immunosuppressive activity *via* upregulation of *STAT3* expression, induction of endoplasmic reticulum stress, as well as utilization or reactive oxygen species (ROS), prostaglandin E_2_, and immunosuppressive cytokines (205, 208). In particular, MDSCs immunosuppressive activity downregulates pivotal signaling pathways, interrupting exploitative pathways utilized by PD-1 immune checkpoint inhibitors *via FATP2* ROS-mediated immunosuppression and upregulated expression of PD-1/PD-L1 secondary to inflammation and T cell exhaustion, which could potentially be reversed *via* MDSC blockade (209–211). Epigenetic modifiers such as HDAC inhibitors have thus been identified to promote sensitization of tumor cells to PD-1/PD-L1 immune checkpoint inhibitors *via* reversal of MDSC’s immunosuppressive effect on the cancer milieu, demonstrating synergistic effects with immunotherapeutic options in solid tumors (212). We observe further agents such as artemisinin used in MDSC suppression, which improved the efficacy of anti-PD-L1 blockade therapy in mice with T cell lymphoma (213). The presence of a deficiency in the MDSC-polarizing *TIPE2* gene additionally informed of MDSC activity and thus delayed tumor progression in mice (214). Though these current studies are of yet relatively removed from the field of PTCL, we believe that these developments can inform clinicians of future aspects for investigation in the field of PTCL surrounding epigenetic and immune checkpoint blockade therapy.

## Conclusions and prospects

**Figure 1:**
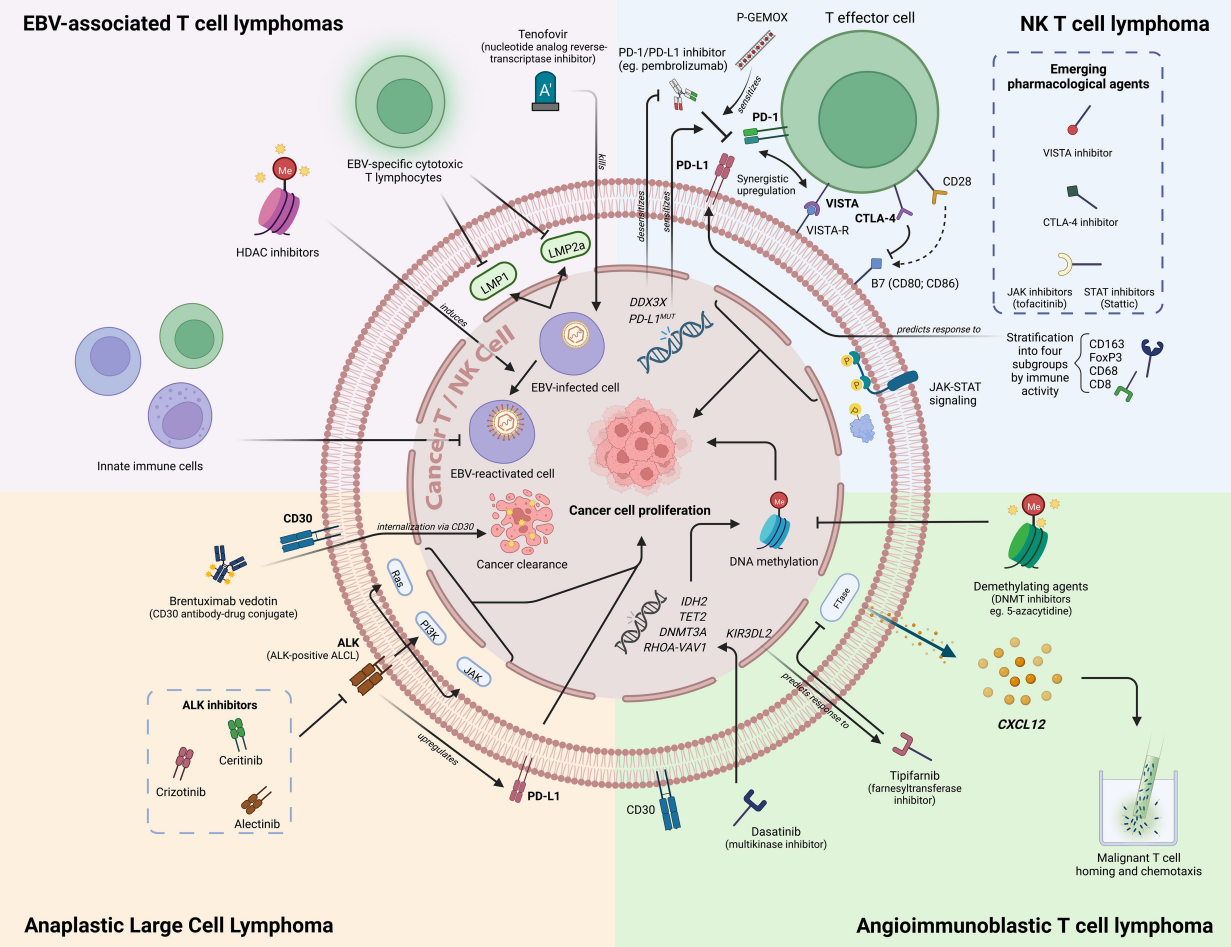
An overview of novel biomarkers in PTCL/NKTCL. Figure created with BioRender.com.

The literature surrounding biomarkers in the exploration of therapeutics in PTCL has come a long way in the last few years with the advent of numerous new trials and studies investigating biomarkers at both the genome and epigenome level. For reference, a consolidated figure delineating the most important biomarkers covered in our Review can be found below under **Figure 1**. Despite this, upon taking a closer look at the literature, we understand that the current landscape of biomarkers includes many that are prognostic in nature, but few and little present further utility as predictive markers to guide real-world clinical therapeutic decisions. A list of specific molecular features with direct effects on patient clinical outcomes and prognosis which can potentially inform clinicians on prognosis and survival into **Table 3** below for reference at a glance.

**Table 3:** Summary of the effect of specific molecular features and biomarkers on prognosis and survival.

Specific Molecular Feature/Biomarker	Effect on Prognosis and Survival	Elaboration on mechanisms	References
Peripheral T cell lymphoma, not otherwise specified (PTCL-NOS)			
GATA3 expression	Reduced 5-year OS of 19% (95CI: 9%-38%) as compared to the TBX-21 subtype	Drives differentiation of mutant T lymphocytes into Th2 subtype Higher burden of chromosomal abnormalities and *MYC* signals Marginal enrichment of β-catenin, mTOR and PI3K gene signatures	Iqbal et al. (38)
TBX21 expression	Improved 5-year OS of 38% (95CI: 25%-56%) as compared to the GATA3 subtype	Drives differentiation of mutant T lymphocytes into Th1 subtype Enhancement of IFNγ and NF-κB gene signatures	
*DNMT3A* mutation	*DNMT3A*-mutant TXB21-positive PTCL-NOS is associated with worse prognosis in CHOP-treated patients, negating the survival benefits of TXB21-positivity	*DNMT3A* mutations in TBX21-positive PTCL-NOS upregulates CD8+ T-cell genes with cytotoxic function	Herek et al. (43)
Angioimmunoblastic T cell lymphoma (AITL)			
PD-1/PD-L1 expression in the tumor environment of AITL	The presence of PD-1/PD-L1 expression in AITL tumor and tumor microenvironment was associated with reduced OS although not statistically significant (p = 0.051)	PD-L1 expression may be induced by EBV, EBV-positivity in AITL might be related to the expression of PD-1/PD-L1 for facilitation in immune evasion	Kim et al. (168)
Anaplastic Large Cell Lymphoma (ALCL)			
ALK-positivity	Favorable prognosis and response to first-line chemotherapy and ALK-targeted therapy	Presence of the genomic arrangement of the ALK gene on chromosome 2, commonly associated with t(2;5)(p23;q35)	Ellin et al. (23)
ALK-negativity	Reduced prognosis and response to first-line chemotherapy as compared to the ALK-positive subtype		Parkhi et al. (57) Parilla et al. (58) Pedersen et al. (59) Hapgood et al. (60)
- *DUSP22* rearrangements	Favorable clinical outcomes with a 5-year OS of 40-90%	The pathways affected by *DUSP22* and *TP63* rearrangements are of yet unidentified; have not yet been exploited for targeted therapeutics	
- *TP63* rearrangements	Poorer clinical outcomes with a 5-year OS of 0-17%		
- ‘triple-negative’	Associated with a 5-year OS of 33-42%	Seen as a heterogenous group with outcomes between that of *DUSP22* and *TP63*-rearranged ALCL	
Extranodal NK/T cell lymphoma, nasal type (NKTCL)			
*DDX3X* mutation	Presence of the *DDX3X* mutation predicts poorer prognosis in patients treated with CHOP-based therapy 2-year OS and PFS rates for subjects with mutated *DDX3X* were 39.4 months (vs 80.8 months in WT *DDX3X*, p < 0.001) and 23.3 months (vs 81.0 months in WT *DDX3X*, p < 0.001)	*DDX3X* regulates RNA helicase and is closely related to the *TP53* tumor suppressor gene Recent studies suggest that *DDX3X* might be the target of *TP53* and could cooperate with TP53 to function as a tumor suppressor	Jiang et al. (65) Wu et al. (215)
EGR1 expression	EGR1 upregulation was consistently identified as able to predict better survival and low-risk patients when correlated *via* assessment using the PINK scoring system for NKTCL	EGR1 is a regulator of multiple genes including *GAS1, CD59, CXCR7, RAMP3*, which are indicators of good prognosis Silencing of EGR1 appears to inhibit cellular apoptosis, chemosensitivity, and radiation-induced apoptosis	Lee et al. (68)
PD-L1 expression	The population with ≥78.2% monocytes in blood with PD-L1 positivity had significantly higher OS and PFS than the population with <78.2% monocytes (p = 0.031 and 0.029 for OS and PFS respectively)	PD-L1/PD-1 interaction which suppresses native T cell activation and adaptive T cell mediated cancer cell clearance	Zhang et al. (116) He et al. (117)
CD38 expression	Strong expression of CD38 in NKTCL was recognized as an independent adverse prognostic factor for PFS (p = 0.009)	CD38 is expressed at lower levels on normal NK cells and can drive activation and proliferation of lymphocytes when activated	Wang et al. (184)
*STAT3* mutation and PRDM1 expression	STAT3 mutation and PRDM1 non-expression were both independently associated with inferior OS (p = 0.017 and 0.037 respectively)	JAK3/STAT3 signaling has been recognized as a discriminating pathway in NK/T cell lymphoma and play significant roles in the pathogenesis of NKTCL	Liu et al. (189)
Primary cutaneous γδ T cell lymphomas (PCGDTL)			
Cell of origin – Vδ1 or Vδ2; epidermis vs dermis vs subcutaneous tissue	Cells of origin from the epidermis (Vδ1) had significantly better survivals (179 months) as compared to those in the dermis (Vδ1, 31 months) and subcutaneous tissue (Vδ2, 12.75 months)	Differences in the cells of origin are hypothesized to contribute to the heterogeneity of clinical presentations, and give rise to unique clinical states	Daniels et al. (76)
EBV-associated PTCLs			
‘Primary EBV-positive nodal T/NK-cell lymphoma’ (PTCL-EBV)	Tumors classified under PTCL-EBV exhibited significantly shorter median OS (4.6 months vs 14.7 months in NKTCL)	These tumors were identified to be clinically distinct from NKTCL, with low genomic instability, upregulation of NK-κB, IFNγ, IL6-JAK-STAT3, and downregulation of EBV miRNA	Myint Wai et al. (202)

OS, overall survival; PFS, progression-free survival; CHOP, chemotherapy regimen consisting of cyclophosphamide, doxorubicin, vincristine, prednisolone; EBV, Epstein-Barr virus; WT, wild-type.

A consolidated table of current novel therapeutics and their efficacies can also be viewed in **Table 4**. Whilst the current climate in the treatment for PTCL sparks optimism in its avid generation of numerous pathways for exploitation *via* novel therapeutic options, the current direction lacks a purpose and an efficiency that can only be matched *via* a better understanding of the efficacy of therapeutics in each individualized patient, guided by predictive biomarkers. There is thus a growing importance to move towards personalized treatment options and individualized targeted therapy as a means of improving treatment outcomes and minimizing mortality profiles. This includes an expansion of the role of prognostic indices to guide decisions in patient selection, along with further updating of our prognostic indices with new data that we have unearthed with respect to the role of molecular and immunological phenotypes in disease prognosis. With this, we can follow up with patient stratification based on assessed immunophenotypic subtypes and profiles for personalized treatment and management.

**Table 4:** Summary of novel therapeutics and data on current efficacies.

Pharmacological mechanism of action	Pharmacological agent	Types of PTCL indicated	Outcomes from studies	References
CD52 cell surface protein inhibitor	Alemtuzumab	Non-specific	Used in combination with first-line CHOP chemotherapy with an ORR of 75% No OS benefit in including alemtuzumab over CHOP-only chemotherapy Significant toxicity and high rate of severe adverse events	Gallamini et al. (93) d’Amore et al. (94) Wulf et al. (95)
CCR4 cell surface protein inhibitor	Mogamulizumab	Non-specific, largely ALK-negative ALCL, PTCL-NOS, AITL	Achieved an ORR of 50% in one study in CCR4+ PTCL Achieved an ORR of 34% in another study, with no significant correlation between CCR4 expression levels and response rates Associated with a high frequency of adverse events, particularly neutropenia and lymphopenia	Remer et al. (98) Ogura et al. (101)
CD30-targeting antibody-drug conjugate	Brentuximab vedotin	Mainly ALCL	Brentuximab vedotin in combination with CHP (BV-CHP) was found to be associated with significantly better outcomes than standard CHOP regimen in CD30-positive (>10% expression) tumors in the first-line setting	Horwitz et al. (78) Horwitz et al. (106)
CD25-targeting fusion protein	Denileukin difitox	Non-specific	Combination chemotherapy with CHOP regimen in treatment naïve patients elicited an ORR of 65% Denileukin difitox monotherapy in R/R PTCL with CD25-positivity (defined as CD25+ expression >10%) attained an ORR of 61.5%; in the population without CD25-positivity ORR was 45.5%	Foss et al. (109) Dang et al. (110)
	E7777	Non-specific	Monotherapy of E7777 achieved an ORR of 41.2%; subgroup analysis revealed the ORR in CD25-positive cells (>20% CD25 expression) to be 41.7% and 25.0% in CD25-negative cells	Kawai et al. (111)
PD-1/PD-L1 immune checkpoint inhibitor	Pembrolizumab	Non-specific	Monotherapy in R/R PTCL achieved an ORR of 33%	Barta et al. (118)
		NKTCL	Monotherapy in NKTCL patients failing first-line L-asparaginase regimens yielded an ORR of 100% with long lasting remission Monotherapy in R/R NKTCL achieved an ORR of 57.1% with an OS and PFS of 5.0 months and 4.8 months respectively	Kwong et al. (121) Li et al. (122)
	Geptanolimab	Non-specific	Monotherapy in R/R PTCL achieved an ORR of 40.4%	Shi et al. (119)
	Nivolumab	Non-specific	Monotherapy in R/R PTCL achieved an ORR of 33%	Bennani et al. (120)
	Avelumab	NKTCL	Monotherapy in R/R NKTCL achieved an ORR of 38%, a CR rate of 24% and response to avelumab was significantly associated with the expression of PD-L1 by tumor tissue (p = 0.001)	Kim et al. (123)
	Sintilimab	NKTCL	Monotherapy in R/R NKTCL achieved an ORR of 75.0% with a 2-year OS of 78.6%	Tao et al. (124)
HDAC inhibitors	Romidepsin	Non-specific	Monotherapy in R/R PTCL achieved an ORR of 25% and a CR rate of 15%	Coiffier et al. (131)
		Non-specific	Combination therapy with oral 5-azacytidine in a population with both treatment-naïve and R/R PTCL achieved an ORR of 61% and CR rate of 43%; subgroup analysis of treatment-naïve patients and T_FH_ patients had an ORR of 70% and 80%	Falchi et al. (134)
		Non-specific	Combination therapy of romidepsin and CHOP failed to achieve significant improvements in OS, PFS or response rates over CHOP-only chemotherapy	Bachy et al. (135)
	Belinostat	Non-specific	Monotherapy in R/R PTCL achieved an ORR of 25.8% and a CR rate of 15.0%	O’Connor et al. (132)
	Chidamide	Non-specific	Monotherapy in R/R PTCL achieved an ORR of 38% and a CR rate of 14%	Shi et al. (133)
		AITL	More durable responses were identified in R/R AITL with an ORR of 50% and CR rate of 40% in this subpopulation	
		NKTCL	Chidamide might have synergistic effects when combined with standard PD-1 blockade therapy in NKTCL	Zhang et al. (125) Wei et al. (126)
DNMT inhibitors	5-azacytidine	AITL	Monotherapy in a cohort comprising almost completely of R/R AITL (except for one patient) achieved an OR of 75% and a CR rate of 50% Monotherapy in R/R AITL achieved an OR of 40% with greater ORR reported in subjects subjected to ≤2 previous chemotherapeutic regimens Monotherapy in R/R AITL yielded a median PFS and OS of 5.4 months and 18.4 months respectively, versus 2.8 months and 10.3 months in a population subjected to investigator’s choice of gemcitabine, bendamustine, or romidepsin. (OS p = 0.0421, PFS HR = 0.557)	Lemonnier et al. (137) Yoon et al. (139) Lemonnier et al. (140)
*JAK* inhibitors	Ruxolitinib	Non-specific	Monotherapy in R/R PTCL achieved CBR of 53% in the cohort with proven activating *JAK* or *STAT* mutations; a CBR of 45% was achieved in the cohort with ≥30% pSTAT3 expression identified *via* immunohistochemistry	Moskowitz et al. (161)
Multikinase inhibitor	Dasatinib	AITL	Targets RHOA-VAV1 signaling in AITL, and in a population of patients which responded to dasatinib 3 of 4 (75%) held a RHOA or VAV1 mutation	Nguyen et al. (166)
Farnesyltransferase enzyme inhibitor	Tipifarnib	AITL	Tipifarnib aids in downregulating *CXCL12* secretion; when trialed in a cohort of *CXCL12*-overexpressed AITL patients, a 43% ORR and 73% CBR was observed Presence of the *KIR3DL2* gene variants additionally predicted complete response to tipifarnib therapy and improved outcomes	Witzig et al. (167)
ALK-targeted inhibitors	Crizotinib	ALK-positive ALCL	Monotherapy achieved a CR rate of 83% (5 of 6 patients) in a pediatric ALK-positive ALCL group Monotherapy achieved an ORR of 90.9% in a heavily pretreated, ALK-positive ALCL group Monotherapy achieved an ORR of 83.3% with a CR rate of 58.3% in a R/R ALK-positive ALCL group	Mosse et al. (170) Gambacorti-Passerini et al. (172) Bossi et al. (173)
	Ceritinib	ALK-positive ALCL	One patient with ALK-positive ALCL achieved complete remission with ongoing clinical benefit after 5 years of therapy	Subbiah et al. (174)
	Alectinib	ALK-positive ALCL	Monotherapy in a population of R/R ALK-positive ALCL yielded an ORR of 80%, with a 1-year OS and PFS of 70.0% and 58.3% respectively	Fukano et al. (175)

OS, overall survival; PFS, progression-free survival; CHOP, chemotherapy regimen consisting of cyclophosphamide, doxorubicin, vincristine, prednisolone; BV-CHP, combination therapy of brentuximab vedotin with chemotherapeutic agents cyclophosphamide, doxorubicin, prednisolone; R/R, recurrent/relapsed; ORR, overall response rate; CBR, clinical benefit rate.

While we work on the identification of relevant tumor markers, we would also do well to continue our investigation into the utility of new molecular techniques. Chimeric antigen receptor (CAR) T cells have been developed as vessels for the deployment of immunologically active treatment towards targeting of malignant T cells in PTCL and show promising activity. Current agents in the market include that of tisagenlecleucel or axicabtagene ciloleucel which are specifically targeted against CD19, but these options have only been evaluated in the setting of B-cell lymphomas (216); CAR-T targeting CD30 has also been trialed in the subset of ALCL in an early phase I study (217). We also consider the possibility of cancer vaccines – vaccines against oncogenic infections such as EBV, but to date no efficacious vaccine development for prophylaxis of EBV infection has been pioneered (218).

Finally, after addressing the above lapses, we would do well to take a slight step back to assess the present roadblocks hindering the feasibility of personalized medicine as a whole. Issues at hand pertaining to the cost of detailed investigations require comprehensive cost-benefit analyses which should inform decision making *via* the stratification of patients into subgroups which predict the utility of further testing. These subgroups should be based on preliminary identification of tumor biology and the selection of high-yield biomarkers for testing, and consistently cross-referenced against present and developing literature. As the throughput for personalized medicine rises, non-invasive techniques such as liquid biopsies could present an important quality of life consideration, utilizing ctDNA as a means for planning for precision therapeutics and serial monitoring of tumor characteristics and biomarker availability. In time, we also envision a role for multiplex methods such as spatial profiling amidst a clinical landscape whereby hundreds of important biomarkers are routinely considered, providing clinicians and researchers alike with the ability to explore disease profiles across space and time. We thus look towards these exciting prospects on the horizon as we continue our work in developing this field towards a refinement of personalized and precision medicine.

## Author contributions

Conceptualization DY, CO, and JC; original draft preparation, DY and JC; writing, review and editing, DY, JL, DH, CO, and JC. All authors contributed to the article and approved the submitted version.
