# Supplementary material for: Emerging predictive biomarkers for novel therapeutics in peripheral T-cell and natural killer/T-cell lymphoma
Source: Front Immunol. 2023 Jan 26;14:1068662. doi: 10.3389/fimmu.2023.1068662 (PMC9909478; doi:10.3389/fimmu.2023.1068662)
Supplement: Supplementary file 1 [file DataSheet_1.docx]

| **General Search Terms** |
| --- |
| (ptcl OR (peripheral T cell lymphoma) OR (Lymphoma, T-Cell, Peripheral[MeSH Terms])) AND (biomarkers OR (Biomarkers[MeSH Terms])) |
| (alcl OR (anaplastic large cell lymphoma) OR aitl OR (angioimmunoblastic t cell lymphoma) OR nktcl OR (NK T cell lymphoma) OR (natural killer T cell lymphoma) AND (biomarkers OR (Biomarkers[MeSH Terms])) |
| **Specific Search Terms** |
| *[Specific Biomarker Term*]* AND (ptcl OR (peripheral T cell lymphoma) OR (Lymphoma, T-Cell, Peripheral[MeSH Terms]))  *Bracket is replaced with specific term such as ‘CD30’, ‘CD52’, etc. |

Annex A – Search Terms
